# Matriptase-2-mediated suppression of hepatic hepcidin expression in mice requires hepatocyte neogenin

**DOI:** 10.1016/j.jbc.2026.111142

**Published:** 2026-01-12

**Authors:** Caroline A. Enns, Shall Jue, An-Sheng Zhang

**Affiliations:** Department of Cell, Developmental, and Cancer Biology, Oregon Health & Science University, Portland, Oregon, USA

**Keywords:** hepatocyte, hepcidin, homeostasis, hormone, iron, matriptase-2, neogenin, serine protease

## Abstract

Neogenin (NEO1) is a ubiquitously expressed multifunctional receptor. It binds members of the repulsive guidance molecules (RGM), RGMa, RGMb, and hemojuvelin (HJV). While RGMa and RGMb binding to NEO1 are necessary for neural development, more recent studies demonstrated that the Neo1-Hjv interaction in hepatocytes plays a pivotal role in iron homeostasis by facilitating hepcidin expression through the bone morphogenetic protein (BMP)-signaling pathway. Hepcidin is an iron regulatory hormone. In mice, ablation of *Neo1* or *Hjv* reduces hepcidin and causes iron overload. Similar effects occur upon disruption of the Neo1-Hjv association. Besides HJV, NEO1 also interacts with matriptase-2 (MT2), a key suppressor for hepcidin expression. MT2 mutations result in an inappropriately high hepcidin and iron-refractory iron-deficiency anemia in humans. MT2 is a membrane-anchored serine protease. It can cleave multiple components of the hepcidin induction pathway *in vitro*, including HJV. In this study, we investigated the roles of Neo1-Mt2 interaction in hepcidin expression *in vivo*. In contrast to the observations that Mt2 cleaves Neo1 and markedly reduces Neo1 levels in cultured hepatoma cells, we found that Mt2 stabilizes Neo1 in murine liver. Studies in mice suggest that Mt2 suppression of hepcidin relies on the presence of Neo1. Additional investigations imply that the major function of hepatic Neo1 is to set the basal levels of hepcidin expression. Together, these data along with the evidence that Mt2 also suppresses the function of Hjv, support the model that Mt2 suppression of hepatic hepcidin is achieved by inhibiting the Neo1/Hjv-induced Bmp-signaling pathway.

Iron is an essential element for life. Either too much or too little iron is detrimental. Since humans cannot excrete excess iron from the body, systemic iron homeostasis is maintained by coordinately regulating the iron absorption in the duodenum, iron recycling from senescent erythrocytes in macrophages, and mobilization of stored iron in the liver (1, 2). Hepcidin, a key iron regulatory hormone, plays an essential role in this process. Hepcidin is 25-amino acid peptide secreted predominantly by hepatocytes. It is encoded by the *HAMP* gene in humans and the *Hamp* gene in mice. Hepcidin controls iron efflux from duodenal epithelial cells, macrophages, and hepatocytes into the circulation by blocking iron export through the only known iron exporter, ferroportin (Fig. 1*A*) (3, 4). Ferroportin is expressed on duodenal enterocytes, macrophages and hepatocytes. Lack of hepcidin causes juvenile hemochromatosis, a severe iron overload disorder (5, 6), whereas inappropriately high levels of hepcidin result in iron-deficiency anemia (1, 2). Under physiological conditions, systemic iron homeostasis is achieved by tight regulation of hepcidin expression in the liver.

**Figure 1:**
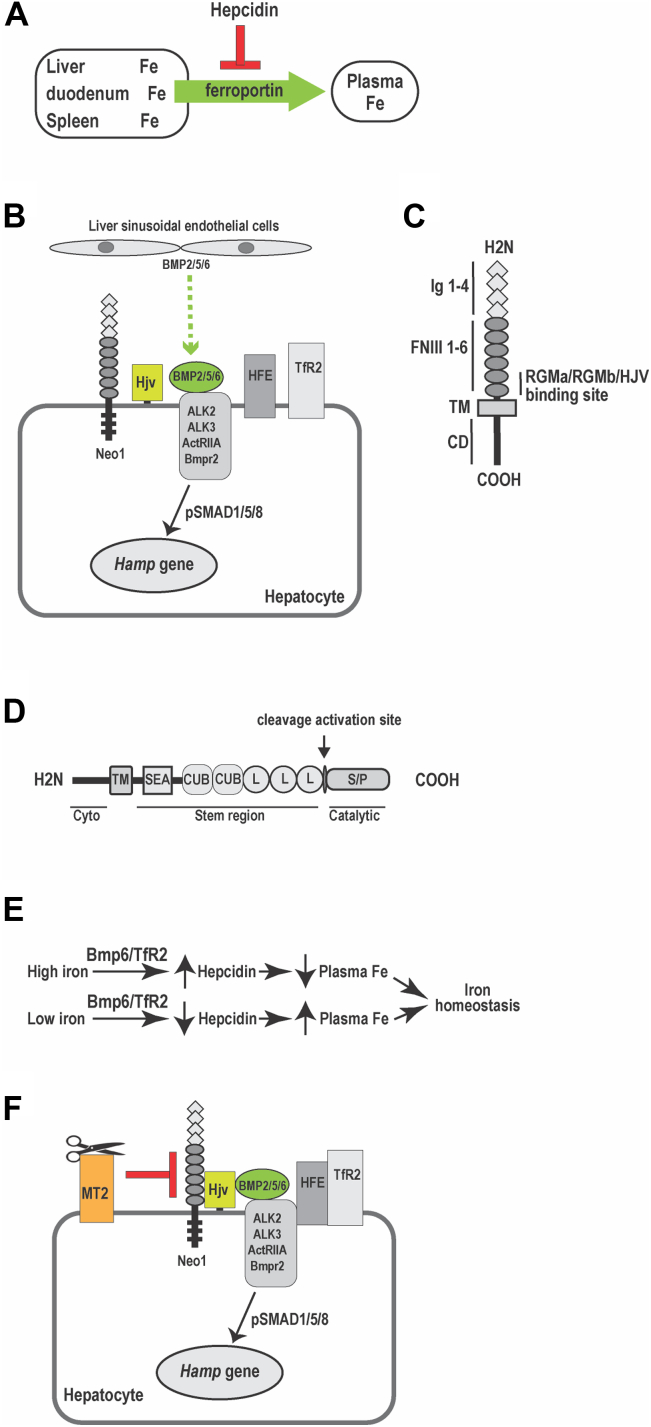
**Hepcidin is an iron regulatory hormone that is secreted mainly by hepatocytes**. *A*, hepcidin inhibits iron efflux from duodenum, spleen, and the liver into the circulation by blocking the plasma-membrane iron exporter, ferroportin. *B*, diagram of the key components that are involved in the induction of hepcidin expression in the liver. *C*, cartoon of NEO1 protein. It contains four immunoglobulin-like (Ig) domain, six fibronectin III domains, a transmembrane domain, and a cytoplasmic domain (CD). *D*, diagram of MT2 protein. Cyto: cytoplasmic domain. transmembranedomain. SEA: sea urchin sperm protein, enteropeptidase agrin. CUB: complement protein subcomponents C1r/C1s, urchin embryonic growth factor and bone morphogenetic protein one domain. L: low-density lipoprotein receptor class-A domain. Catalytic: serine protease (S/P) catalytic domain. The *arrow* indicates the predicted autocleavage activation site. *E*, iron induction of hepcidin expression by increases in *Bmp6* gene transcription and TfR2 protein stabilization. *F*, a proposed model for the function of MT2. NEO1, neogenin.

Hepatic hepcidin expression is induced *via* the BMP-signaling pathway (7, 8, 9), which utilizes a selective set of BMP ligands (BMP2, BMP5, and BMP6), BMP receptors (ALK2, ALK3, ActRIIA, and BMPR2), and cytoplasmic SMADs (SMAD1/5/8) (Fig. 1*B*) (7, 8, 9, 10, 11, 12, 13, 14, 15, 16, 17, 18, 19, 20). In mice, the essential BMP ligands, Bmp2/5/6, are derived from liver sinusoidal endothelial cells (13, 14, 15, 16, 17, 19, 20). Importantly, a normal range of hepcidin expression also requires other plasma membrane proteins including neogenin (Neo1), hemojuvelin (Hjv), hemochromatosis protein (Hfe), and transferrin receptor-2 (Tfr2) (1, 21, 22). Global or hepatocyte-specific ablation of any one of these genes reduces hepcidin expression and results in iron overload (22, 23, 24, 25, 26, 27). In humans, specific mutations in the *HFE*, *HJV*, or *TfR2* gene cause hemochromatosis (6, 21, 28).

NEO1 is a ubiquitously expressed multifunctional receptor that binds members of the repulsive guidance molecules, RGMa, RGMb, and HJV (Fig. 1*C*) (29, 30, 31, 32, 33, 34, 35). RGMa and RGMb are mainly expressed in the developing nervous system, and are necessary for neural development (36). Like the other RGMs, HJV is a glycosylphosphatidylinositol-linked membrane protein. Unlike the other RGMs, HJV is predominantly expressed in hepatocytes, skeletal muscle cells, and cardiomyocytes. Animal studies demonstrate that only the hepatic Hjv is indispensable for iron homeostasis (23, 24). Hepatocyte HJV acts as a co-receptor for BMP6 and uses ALK2 and ALK3 to robustly stimulate hepcidin expression *via* the BMP-signaling pathway (8, 10, 16, 17, 37). *In vivo* studies reveal that the induction of hepcidin by Neo1 and Hjv depends on their interaction (22, 30). Structural studies show that HJV can simultaneously bind NEO1 and BMP ligand or ALK3 (34, 35). ALK3 is a pivotal type-I BMP receptor for hepcidin expression (10). Additional investigations indicate that hepatic hepcidin expression also depends on the HFE/TfR2 interaction (38, 39), the HFE/ALK3 interaction (40), the NEO1/ALK3 interaction (30, 41), and the formation of HFE/TfR2/HJV complex (42). Thus, the induction of hepcidin expression by these components is likely achieved by forming a membrane-bound complex in hepatocytes to enhance the BMP-signaling.

Balanced iron homeostasis also requires matriptase-2 (MT2), a key negative regulator of hepcidin expression (13, 21, 43, 44). MT2 is a type-II transmembrane (TM) serine protease that is expressed predominantly in hepatocytes (45). It is composed of a short N-terminal cytoplasmic domain, a transmembrane domain, and an extracellular domain, which contains a membrane-proximal stem region, a predicted activation domain, and a C-terminal catalytic domain (Fig. 1*D*) (45). MT2 is encoded by the *TMPRSS6* gene in humans and the *Tmprss6* gene in mice (45). Mutations in MT2 result in inappropriately high hepcidin expression leading to iron-refractory iron-deficiency anemia (21, 46, 47). Similar phenotypes and alopecia (an indicator of low iron levels in the body) are also found in *Tmprss6*^*−/−*^ mice (48, 49, 50, 51). MT2 suppresses hepcidin expression by reducing the BMP-signaling (1, 2), and the major function of Mt2 in iron homeostasis is to set hepatic hepcidin expression to an appropriate level (52). The underlying mechanism remains controversial. In cultured cells, MT2 cleaves multiple components of the hepcidin-inducing pathway, including HJV, Bmp receptors (Alk2, Alk3, ActRIIA, and Bmpr2), Hfe, and Tfr2, and reduces their levels in the cells (29, 51, 53, 54). These observations suggest that MT2 inhibition of hepcidin expression is mediated through its proteolytic activity. However, studies in mice indicate that Mt2 down-regulates hepcidin through a nonproteolytic mechanism by association with its binding partners to block their functions (52, 55).

MT2 interacts with HJV and NEO1 (29, 30, 53). Since mice deficient for both *Tmprss6* and *Hjv* display a phenotype of *Hjv*^*−/−*^ mice with a marked reduction of hepcidin and severe iron overload (50, 56), it is generally thought that Hjv is the target of Mt2. However, additional investigation reveals that increased Mt2 in the liver of *Hjv*^*−/−*^ mice is able to further suppress hepcidin in the absence of Hjv (51). This observation suggests that Mt2 suppresses hepcidin expression by acting on Hjv as well as other undefined binding partners in the liver.

The liver possesses an elegant but not fully defined machinery to positively regulate hepcidin expression in response to bodily iron load *via* the BMP-signaling pathway to maintain iron homeostasis (Fig. 1*E*) (2, 13, 21, 57, 58, 59, 60, 61). Increased BMP-signaling elevates hepcidin production to limit iron efflux from duodenal epithelial cells, macrophages, and hepatocytes into the circulation by inhibiting the function of ferroportin (3, 4). Hepatic Tfr2 is stabilized by iron-saturated transferrin (62, 63), and *Bmp2* and *Bmp*6 expression in the liver are transcriptionally elevated by increased liver iron storage (7, 16, 17, 20, 64). Tfr2 and Bmp2/6 are, therefore, thought to be the sensors for iron in the circulation and in storage, respectively. It is not known whether hepatic Neo1 is involved in this iron sensing mechanism.

In this study, we tested the hypothesis that hepatic Neo1 is another key target of Mt2 in addition to Hjv by using hepatoma cells and mice as models (Fig. 1*F*). Our results indicate that Mt2 suppression of hepcidin requires Neo1 and suggest that the major function of Neo1 is to set the basal levels of hepcidin expression. Additionally, our results also imply that the Hep3B cell line is not a good model to study the physiological function of Neo1.

## Results

### Mt2 expression markedly reduces Neo1 levels in hepatoma cells

**Figure 2:**
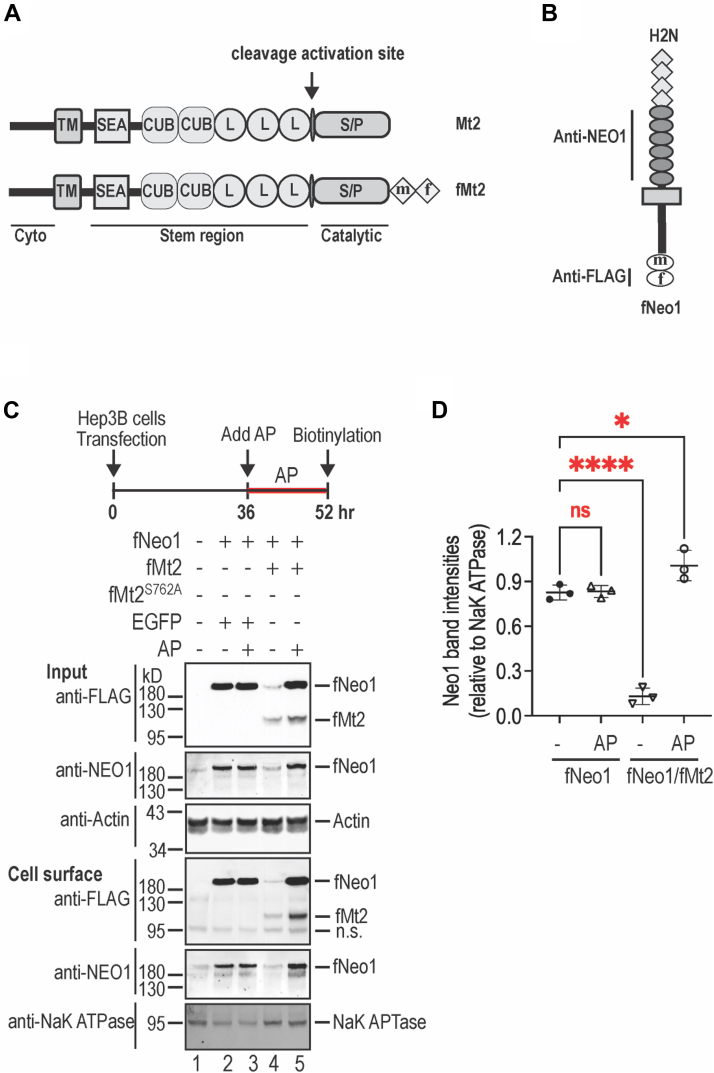
**Mt2 expression markedly reduces Neo1 levels in Hep3B cells**. *A*, diagram of Mt2 and fMt2. The MYC (m) and FLAG (f) epitopes were added to the C-terminus of Mt2. *B*, diagram of fNeo1. The MYC (m) and FLAG (f) epitopes were added to the C-terminus of Neo1. *C*, coexpression of fMt2 with fNeo1 markedly reduces fNeo1 levels in whole cell extracts (input) and on cell surface by biotinylation. AP, aprotinin. Experiments were repeated three times with consistent results. *D*, quantification of cell surface fNeo1 bands in *C* (*panel 4*). The relative amounts to Na + K + ATPase (*panel-5*) are presented (n = 3). The data shown are means ± SD. One-way ANOVA was used to analyze the data. ns, no statistical difference. ∗, *p* < 0.05; ∗∗∗∗, *p* < 0.0001. NEO1, neogenin.

To seek insight into the roles of NEO1-MT2 interaction in hepcidin expression, we first examined the effect of Mt2 on Neo1 in Hep3B cells, a hepatoma cell line that endogenously expresses low levels of *NEO1*, but not *TMPRSS6* mRNA (22, 55). Both murine Neo1 and Mt2 constructs with a C-terminal FLAG/MYC epitope (fNeo1 and fMt2; Fig. 2, *A* and *B*) were transiently co-transfected as previously described (29, 52). Addition of a C-terminal FLAG/MYC epitope does not affect the function of Neo1 or Mt2 when expressed in the liver (22, 55). As shown in Figure 2*C*, expression of fMt2 was able to markedly reduce fNeo1 levels in whole cell extracts and on the cell surface by anti-NEO1 and anti-FLAG antibodies (panel 1/2/4/5, lane 2 vs 4). Since this reduction was completely abrogated by aprotinin (Fig. 2*C*, lane 4 vs 5; Fig. 2*D*), a serine protease inhibitor, these results indicate that fMt2 can specifically cleave fNeo1 in Hep3B cells, consistent with the earlier co-transfection studies for other hepcidin-inducing components in cultured cells (29, 51, 53, 54).

### Ablation of Tmprss6 fails to increase Neo1 levels in the liver

**Figure 3:**
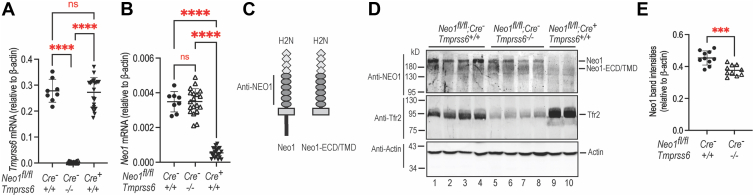
**Ablation of Tmprss6 does not increase Neo1 levels in the liver**. *A* and *B*, qRT-PCR analysis of *Tmprss6* and *Neo1* mRNA in the liver of 8-weeks old *Neo1*^*fl/fl*^;*Alb-Cre*^*-*^;*Tmprss6*^+/+^, *Neo1*^*fl/fl*^;*Alb-Cre*^*-*^;*Tmprss6*^−/−^, and *Neo1*^*fl/fl*^;*Alb-Cre*^*+*^;*Tmprss6*^+/+^ mice. All mice were fed a standard diet containing 240-ppm iron. Each group consists of at least eight mice with similar numbers of male and female. Results are expressed as the amount relative to that of *β-actin* for each sample. The mean values and the standard deviation (SD) are presented. One-way ANOVA was used to analyze the data relative to WT (*Neo1*^*fl/fl*^;*Alb-Cre*^*-*^;*Tmprss6*^+/+^) mice. ns, no statistical difference; ∗∗∗∗, *p* < 0.0001. *C*, diagram of the full-length Neo1 (Neo1), the truncated Neo1 that lacks the intracellular domain (Neo1-ECD/TMD), as well as the anti-human NEO1 antibody used for Western blot analysis. ECD, ectodomain. TMD, transmembrane domain. *D*, western blot analysis of Neo1, Tfr2, and β-actin in the liver membrane preparations from the mice in *A* or *B*. *E*, quantification of full-length Neo1 bands in *D*. The relative amounts to β-actin are presented. Two-tailed student *t* test was also used for analysis. ∗∗∗, *p* < 0.001. NEO1, neogenin.

Since Hep3B cells are a relatively undifferentiated hepatoma cell line and do not express many of the genes that are involved in iron homeostasis (22), we next examined the impact of Mt2 on Neo1 in murine liver by comparing the hepatic Neo1 levels in eight-week-old WT and *Tmprss6*^−/−^ mice. The hepatocyte-specific *Neo1* knockout (*Neo1*^*fl/fl*^;*Alb-Cre*^*+*^) mice were included as a control. We reasoned that if Mt2 cleaves Neo1 *in vivo*, lack of Mt2 would cause an increase in hepatic Neo1. As shown in Figure 3, *A* and B, ablation of *Tmprss6* gene does not affect *Neo1* mRNA levels in the liver, and ablation of hepatic *Neo1* does not significantly alter the levels of *Tmprss6* mRNA. In agreement with earlier reports (48, 49, 50, 52), both genders of *Tmprss6*^*−/−*^ mice displayed alopecia, microcytic anemia, and low serum iron because of the inappropriately high hepatic *hepcidin* (*Hamp*) mRNA levels (Fig. S1, *A*–*E*). High hepcidin blocks dietary iron absorption by directly occluding the plasma membrane iron exporter, ferroportin, as well as by inducing its internalization and degradation (Fig. 1*A*) (3, 4). Owing to the relatively low levels of *Neo1* expression (Fig. 3*B*), the liver membrane fractions were prepared for Western blot analysis. Consistent with our earlier studies, two different Neo1 bands were detected in the liver membrane preparations of WT mice by using an anti-human NEO1 antibody (Fig. 3, *C* and *D*, top panel, lane 1–4). The top band represents the full-length Neo1tr, and the lower band is the γ-secretase-cleaved form of Neo1 that lacks the intracellular domain (Neo1-ectodomain/TMD) (22, 41). Interestingly, although the relative *Tmprss6* mRNA levels were much higher than those of Neo1 mRNA in the liver of WT mice (Fig. 3, *A* and *B*), ablation of *Tmprss6* did not significantly increase Neo1 levels in the liver membrane preparations (Fig. 3*D*; top panel, lane 1–4 vs 5–8). Rather we observed a modest but significant decrease in Neo1 (Fig. 3*E*). The specificity for anti-NEO1 antibody was validated by the marked reduction of Neo1 in *Neo1*^*fl/fl*^;*Alb-Cre*^*nbsp;+*^ mice (Fig. 3*D*; lanes 9–10). Since hepatic hepcidin expression is induced by the full-length Neo1, but not the truncated Neo1-ectodomain/TMD, in our earlier studies (41), only the full-length Neo1 bands were analyzed in this and the following studies. Consistent with the iron deficiency in *Tmprss6*^*−/−*^ mice (48, 49, 50, 51), hepatic Tfr2 protein levels are markedly decreased by the low serum iron concentrations (Fig. 3*D*; panel-2, lanes 1–8; Fig. S1*F*). Hepatic Tfr2 is stabilized by iron-saturated transferrin in the circulation (62, 63). Together, these results suggest that Mt2 stabilizes, rather than cleaves, Neo1 in the liver and imply that Mt2 acts differently *in vivo*.

### Expression of exogenously administered Mt2 in the liver of Tmprss6^−/−^ mice increases Neo1 levels

**Figure 4:**
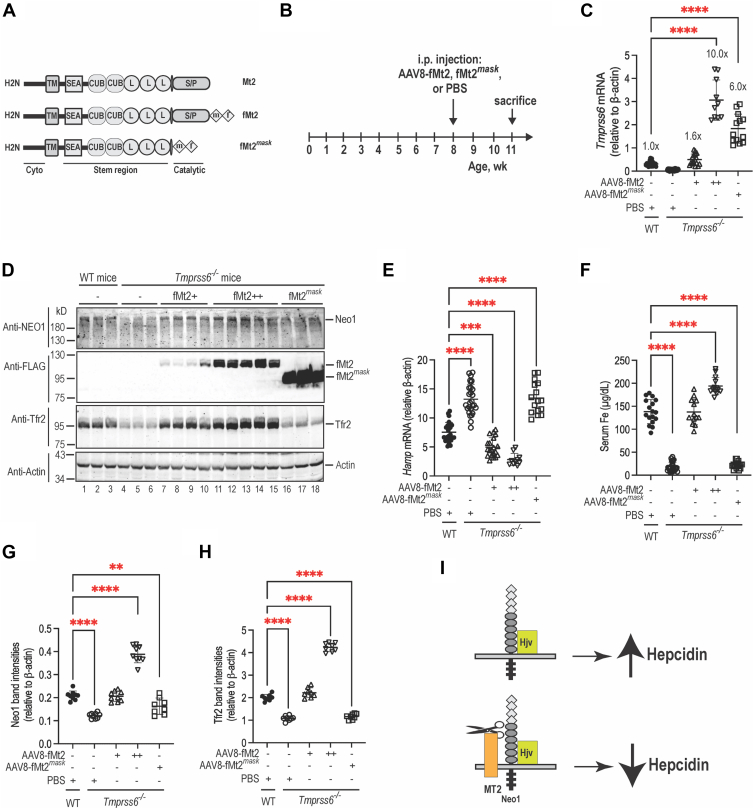
**Expression of exogenously administered Mt2 in the liver of Tmprss6^−/−^ mice increases Neo1 levels**. *A*, diagram of Mt2, fMt2, and fMt2^mask^. *B*, experimental design to determine the effects of exogenously administered Mt2 on Neo1 levels. Eight-week-old *Tmprss6*^*−/−*^ littermates on a mixed B6/129 background were intraperitoneally injected with AAV8-fMt2 vectors at ∼8 x 10^11^ or ∼4 x 10^12^ viral genome-particles per mouse or AAV8-fMt2^mask^ viral vectors at ∼4 x 10^12^ viral genome-particles per mouse. Injection of sterilized PBS vehicle was included as a control. Age and gender-matched WT littermates were included as additional controls. Mice were euthanized for analysis 3 weeks after injection. All mice were fed a standard diet containing 240-ppm iron. Each group consists of at least five mice with similar numbers of male and female. *C*, qRT-PCR analysis of *Tmprss6* mRNA in the liver. Results are expressed as the amount relative to that of *β-actin* for each sample. The mean values and SD are presented. *D*, representative images of Western blot analysis for Neo1, the FLAG epitope of fMt2, Tfr2, and β-actin in the liver membrane preparations. *E*, qRT-PCR analysis of *Hamp* mRNA in the liver. *F*, serum iron assay. *G*,. quantification of Neo1 bands in *D*. *H*, quantification of Tfr2 bands in *D*. The relative amounts to β-actin are presented. One-way ANOVA was used to analyze the data relative to WT mice. ∗∗, *p* < 0.01; ∗∗∗, *p* < 0.001; ∗∗∗∗, *p* < 0.0001. *I*, models for Neo1/Hjv induction of hepcidin expression in the absence and presence of Mt2. NEO1, neogenin.

To determine whether Mt2 suppression of hepcidin expression requires its proteolytic activity, we examined the effects of fMt2 on endogenous Neo1 in the liver. Two different levels of fMt2 were expressed in the hepatocytes of *Tmprss6*^−/−^ littermates on a mixed B6/129S background *via* AAV8 viral vectors (51, 54, 55, 65). fMt2^*mask*^ that lacks the proteolytic domain (Fig. 4*A*) was included as a negative control (51, 54). This AAV8 vector specifically expresses the gene of interest in hepatocytes, and the transduced gene is evenly expressed in hepatocytes throughout the liver (66), similar to the homogenous distribution of native *Tmprss6* mRNA in hepatocytes (13). Administration of AAV8 vector alone had no effect on iron homeostasis in mice (51, 54). Animals were euthanized at ∼11 weeks old for analysis (Fig. 4*B*). Results show that the fMt2 mRNA levels (Fig. 4*C*) are proportional to the protein levels by Western blot analysis using an anti-FLAG antibody (Fig. 4*D*; panel-2, lanes 7–15). The inappropriately high fMt2^*mask*^ protein levels (Fig. 4*D*; panel-2, lanes 16–18) are likely due to its higher stability (51, 54). Consistent with our previous studies (51, 54, 55), expression of exogenous fMt2 mRNA at the levels comparable to WT counterparts was able to fully correct the inappropriately high hepcidin expression and low serum iron status (Fig. 4, *C* and *E*, *F*), and greatly ameliorated the anemia when compared with PBS or AAV8-fMt2^*mask*^-injected *Tmprss6*^−/−^ controls (Fig. S2). Expression of ∼10-fold higher fMt2 mRNA than WT mice was able to further reduce *Hamp* mRNA levels (Fig. 4, *C*–*E*).

Similar to the observations in 8-week-old *Tmprss6*^−/−^ mice (Fig. 3, *D* and *E*), we also detected a significant decrease in hepatic Neo1 in 11-week-old *Tmprss6*^−/−^ mice (Fig. 4, *D* and *G*). Interestingly, this decrease was fully corrected upon expression of exogenous fMt2. Importantly, the increases in Neo1 levels are positively associated with those of fMt2 (Fig. 4, *D* and *G*). In comparison, only a modest increase in Neo1 was observed when higher levels of fMt2^*mask*^ were expressed (Fig. 4, *D* and *G*). These results further indicate that Mt2 acts to stabilize Neo1 *in vivo* and suggest that Hep3B cells are not an appropriate model to study the function of Mt2. Additionally, we examined the changes of hepatic Tfr2 and found that Tfr2 levels are also increased by fMt2 (Fig. 4, *D* and *H*). Earlier studies show that Mt2 cleaves and reduces Tfr2 in transfected cells (51) and that Tfr2 is stabilized by increased Tf saturation (62, 63). Since the Tfr2 levels are positively correlated with serum iron concentrations (Fig. 4, *F* and *H*), we speculate that the fMt2-mediated increases in Tfr2 likely result from holo-transferrin-mediated stabilization. In conjunction with the observation that increased fMt2 does not alter Hjv levels in the liver (55), these data suggest that Mt2 suppression of hepcidin expression is not achieved through its proteolytic activity and support the idea that Mt2 binds and sequesters its binding partners to inhibit their participation in iron homeostasis (Fig. 4*I*).

### Overexpression of Mt2 in the liver of WT mice does not reduce Neo1

**Figure 5:**
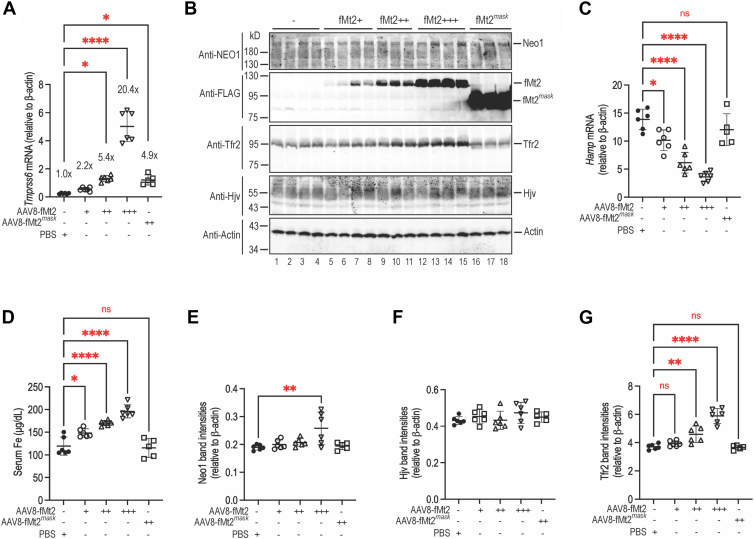
**Increased Mt2 expression in the liver of WT mice does not reduce Neo1**. Eight-week-old 129S mice were intraperitoneally injected with AAV8-fMt2 vectors at ∼8 x 10^11^, ∼3 x 10^12^, or ∼6 x 10^12^ viral genome-particles per mouse or AAV8-fMt2^mask^ viral vectors at ∼4 x 10^12^ viral genome-particles per mouse. Injection of sterilized PBS vehicle was included as a control. Mice were euthanized for analysis 3 weeks after injection. All mice were fed a standard diet containing 240-ppm iron. Each group consists of at least five mice with similar numbers of male and female. *A*, qRT-PCR analysis of *Tmprss6* mRNA in the liver. Results are expressed as the amount relative to that of *β-actin* for each sample. The mean values and SD are presented. *B*, representative images of Western blot analysis for Neo1, the FLAG epitope of fMt2, Tfr2, Hjv, and β-actin in the liver membrane preparations. *C*, qRT-PCR analysis of *Hamp* mRNA in the liver. *D*, serum iron assay. *E*–*G*, quantification of Neo1, Hjv, and Tfr2 bands in *B*. The relative amounts to β-actin are presented. One-way ANOVA was used to analyze the data relative to PBS-injected WT controls. ns, no statistical difference. ∗, *p* < 0.05; ∗∗, *p* < 0.01; ∗∗∗∗, *p* < 0.0001. NEO1, neogenin.

To seek further insights into the mechanism by which Mt2 suppresses hepcidin, we tested the effects of fMt2 overexpression on hepatic Neo1 levels in WT 129S mice. Three different dosages of AAV8-fMt2 viral vectors were administered intraperitoneally as previously described (51, 55). Animals were euthanized for analysis 3 weeks after injection as depicted above for *Tmprss6*^−/−^ mice (Fig. 4*B*). Results revealed that the total Mt2 mRNA levels in the liver reached about 2.2, 5.4, and 20.4-fold higher than those of endogenous Mt2 mRNA in PBS-injected control group (Fig. 5*A*). The expressed fMt2 protein levels were proportional to the mRNA levels by Western blot analysis using an anti-FLAG antibody (Fig. 5*B*; panel-2, lanes 5–15). Consistent with our previous studies (51), increased fMt2 expression was able to further decrease hepatic *Hamp* mRNA levels with concomitant increases in serum iron concentrations (Fig. 5, *C* and *D*). These data indicate that Mt2 is a limiting factor in iron homeostasis. No significant decreases in hepatic Neo1 or Hjv were detected by increased fMt2 when compared with fMt2^*mask*^ controls (Fig. 5, *B* and *E*, *F*). Rather, we observed a trend increase in Neo1 in the high fMt2 group as well as the fMt2-dependent increases in Tfr2 and serum iron concentrations (Fig. 5, *B* and *D*, *E*, *G*). The specificity for anti-Hjv antibody was validated in our previous studies (55). There is no commercial antibody that can sensitively immunodetect endogenous Hjv. These data further support that Mt2 suppression of hepcidin is not mediated by cleavage of its binding partners.

### Simultaneous ablation of both Neo1 and Tmprss6 in the liver causes iron overload in mice

**Figure 6:**
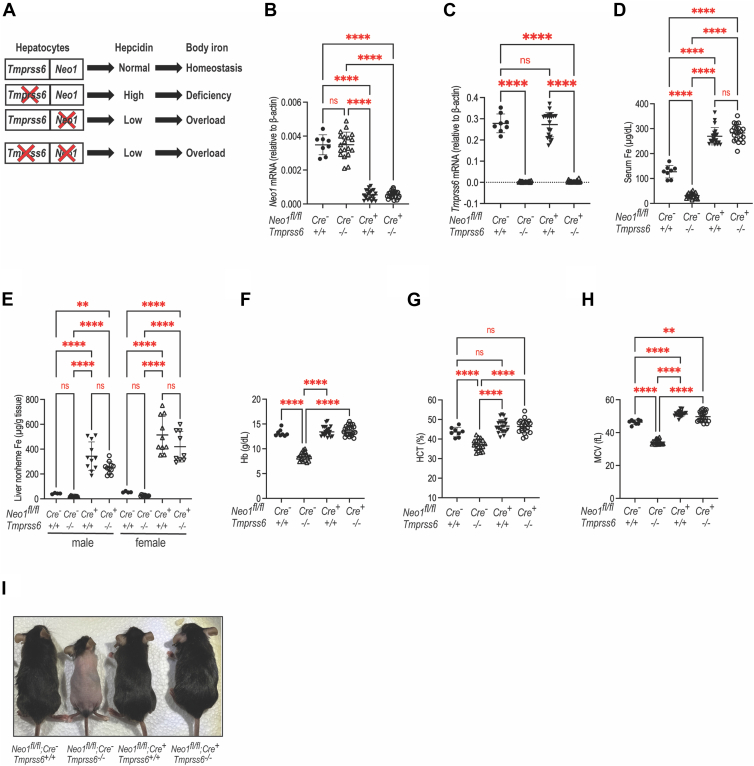
**Ablation of both Tmprss6 and hepatic Neo1 causes iron overload in mice**. *A*, diagram for the possible consequences in mice with ablation of both *Tmprss6* and hepatic *Neo1*. *B* or *C*, qRT-PCR analysis of *Neo1* and *Tmprss6* mRNA levels in the liver of 8-week-old *Neo1*^*fl/fl*^;*Alb-Cre*^*-*^;*Tmprss6*^+/+^, *Neo1*^*fl/fl*^;*Alb-Cre*^*-*^;*Tmprss6*^−/−^, *Neo1*^*fl/fl*^;*Alb-Cre*^*+*^;*Tmprss6*^+/+^, and *Neo1*^*fl/fl*^;*Alb-Cre*^*+*^;*Tmprss6*^−/−^ mice. All mice were fed a standard diet containing 240-ppm iron. Each group consists of at least eight mice with similar numbers of male and female. Results are expressed as the amount relative to that of *β-actin* for each sample. *D*, serum iron assay. *E*, liver non-heme iron assay. Data for male and female animals are separately presented. *F*–*H*, blood parameters: hemoglobin, hematocrit, and mean corpuscular volume. All data shown are means ± SD. One-way ANOVA was used for analysis. ns, no statistical difference. ∗∗, *p* < 0.01; ∗∗∗∗, *p* < 0.0001. *I*, a representative image of mice at the time of euthanasia. NEO1, neogenin.

To determine the roles of Neo1-Mt2 interaction in iron homeostasis, we generated *Neo1*^−/−^;*Tmprss6*^−/−^ mice with ablation of both *Neo1* and *Tmprss6* in hepatocytes (*Neo1*^*fl/fl*^;*Alb-Cre*^*+*^;*Tmprss6*^−/−^). We reasoned that if *Neo1*^−/−^;*Tmprss6*^−/−^ double mutant mice have low hepcidin and iron overload similarly to the *Neo1*^−/−^ single mutant (*Neo1*^*fl/fl*^;*Alb-Cre*^*+*^;*Tmprss6*^+/+^), it would indicate that Mt2 suppression of hepcidin relies on Neo1 (Fig. 6*A*). Conversely, if *Neo1*^−/−^;*Tmprss6*^−/−^ double mutant mice displayed inappropriately high hepcidin and iron deficiency analogously to *Tmprss6*^−/−^ mice (*Neo1*^*fl/fl*^;*Alb-Cre*^*-*^;*Tmprss6*^−/−^), it would suggest that Mt2 suppresses hepcidin independently of Neo1. We first examined the bodily iron status in *Neo1*^−/−^;*Tmprss6*^−/−^ mice and compared with those in *Tmprss6*^−/−^, *Neo1*^−/−^, and WT (*Neo1*^*fl/fl*^;*Alb-Cre*^*-*^;*Tmprss6*^+/+^) littermates. All animals were fed a rodent diet containing 240-ppm iron after weaning and analyzed at 8 weeks old. The *Neo1* and *Tmprss6* ablation in the liver was confirmed by qRT-PCR (Fig. 6, *B* and *C*). Consistent with our earlier studies (22), ablation of hepatocyte *Neo1* alone (*Neo1*^−/−^ mice) resulted in iron overload as manifested by marked increases in serum iron concentrations and liver nonheme iron levels when compared with the corresponding WT controls (Fig. 6, *D* and *E*). The liver nonheme iron is widely used as an indicator for bodily iron load. Interestingly, ablation of both *Neo1* and *Tmprss6* (*Neo1*^−/−^;*Tmprss6*^−/−^ mice) completely abrogated all the iron deficient defects seen in *Tmprss6*^−/−^ littermates, including low serum iron, low liver nonheme iron, low hemoglobin, low hematocrit, low mean corpuscular volume, alopecia, and lower body weight (Fig. 6, *D*–*I*; Fig. S3). Rather, the *Neo1*^−/−^;*Tmprss6*^−/−^ mice display a comparable extent of iron overload as seen in *Neo1*^−/−^ counterparts (Fig. 6, *D* and *E*). These observations indicate that the function of Mt2 in iron homeostasis requires the expression of Neo1 in hepatocytes.

### Mt2 suppression of hepcidin expression is mediated largely by inhibition of Neo1-facilitated Bmp-signaling

**Figure 7:**
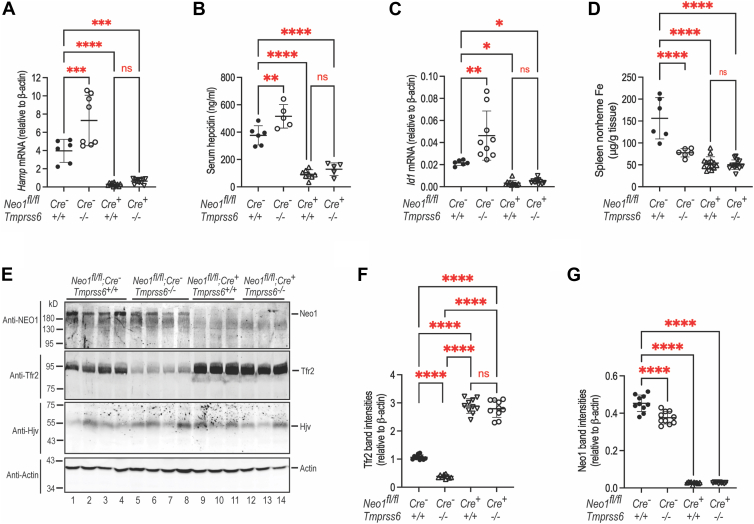
**Ablation of both Tmprss6 and hepatic Neo1 reduces hepatic hepcidin expression**. *A*, qRT-PCR analysis of *Hamp* mRNA in the liver of 8-week-old male *Neo1*^*fl/fl*^;*Alb-Cre*^*-*^;*Tmprss6*^+/+^, *Neo1*^*fl/fl*^;*Alb-Cre*^*-*^;*Tmprss6*^−/−^, *Neo1*^*fl/fl*^;*Alb-Cre*^*+*^;*Tmprss6*^+/+^, and *Neo1*^*fl/fl*^;*Alb-Cre*^*+*^;*Tmprss6*^−/−^ mice as described in the legend to Figure 6. *B*, serum hepcidin assay of male mice. *C*, qRT-PCR analysis of *Id1* mRNA in the liver of male mice. *D*, spleen nonheme iron assay of male mice. *E*, representative images of Western blot analysis for Neo1, Tfr2, Hjv, and β-actin in the liver membrane preparations. Panel-1, 2, and four were cropped from the same set of Western blot images as illustrated in Figure 3*D*. *F*, quantification of Tfr2 bands in *E*. *G*, quantification of Neo1 bands in *E*. The relative amounts to β-actin are presented. All results are expressed as the amount relative to that of *β-actin* for each sample. All data shown are means ± SD. One-way ANOVA was used for analysis. ns, no statistical difference. ∗, *p* < 0.05; ∗∗, *p* < 0.01; ∗∗∗, *p* < 0.001; ∗∗∗∗, *p* < 0.0001. NEO1, neogenin.

We next determined whether the increased iron load in *Neo1*^−/−^;*Tmprss6*^−/−^ mice results from reduced Bmp-signaling and hepcidin expression by using the animals as described above in Figure 6. In agreement with earlier studies (48, 49, 50, 52), the inappropriately high hepcidin expression in *Tmprss6*^−/−^ mice resulted from an elevated Bmp-signaling as indicated by increased hepatic *Inhibitor of DNA binding-1* (*Id1*) mRNA levels when compared with the corresponding WT controls (Fig. 7, *A*–*C*; Fig. S4, *A*–*C*). *Id1* is a direct downstream target of Bmp-signaling pathway, and the levels of *Id1* mRNA are widely used as a sensitive indicator for the status of Bmp-signaling. These results indicate that in the presence of hepatic Neo1, Mt2 suppresses hepcidin expression by inhibiting Bmp-signaling. Consistent with our previous studies (22), lack of hepatic *Neo1* in *Neo1*^−/−^ mice led to reduced Bmp-signaling, decreased hepatic *Hamp* mRNA and serum hepcidin, and decreased spleen iron levels (Fig. 7, *A*–*D*; Fig. S4, *A*–*D*). Since lack of hepatic Neo1 did not significantly reduce the expression levels of the key hepcidin-inducing components in the liver (22), these data are consistent with the idea that hepatic Neo1 is an essential inducer of hepcidin expression by enhancing the Bmp-signaling. Interestingly, a simultaneous lack of both hepatic *Neo1* and *Tmprss6* in *Neo1*^−/−^;*Tmprss6*^−/−^ mice resulted in comparable extents of decreases in hepatic *Hamp* mRNA, serum hepcidin, *Id1* mRNA, and spleen nonheme iron as seen in the *Neo1*^−/−^ littermates (Fig. 7, *A*–*D*; Fig. S4, *A*–*D*). Notably, in the absence of hepatic *Neo1*, ablation of *Tmprss6* was unable to significantly increase *Hamp* mRNA, serum hepcidin, and *Id1* mRNA (Fig. 7, *A*–*C*; Fig. S4, *A*–*C*). No significant decreases in Hjv protein or *Alk2*, *Alk3*, *Hfe*, *Tfr2*, *and Bmp6* mRNA levels were detected (Fig. 7E; Fig. S4, *E*–*J*). These observations indicate that lack of hepatic Neo1 (Fig. 7, *E* and *G*) significantly abrogates Mt2 suppression of Bmp-signaling and hepcidin expression and suggest that Mt2 suppression of hepcidin expression is achieved largely by inhibiting Neo1-facilitated Bmp-signaling.

Additionally, we detected similar degree of increased Tfr2 protein and comparable trend increases in *Bmp6* mRNA levels in response to elevated serum iron and liver nonheme iron in *Neo1*^−/−^ and *Neo1*^−/−^;*Tmprss6*^−/−^ mice (Fig. 6, *D* and *E* & 7, *E* and *F*; Fig. S4*J*). These results suggest that *Neo1*^−/−^;*Tmprss6*^−/−^ mice retain an intact iron sensing mechanism.

### Hepatic Neo1 sets the basal levels of hepatic hepcidin expression

**Figure 8:**
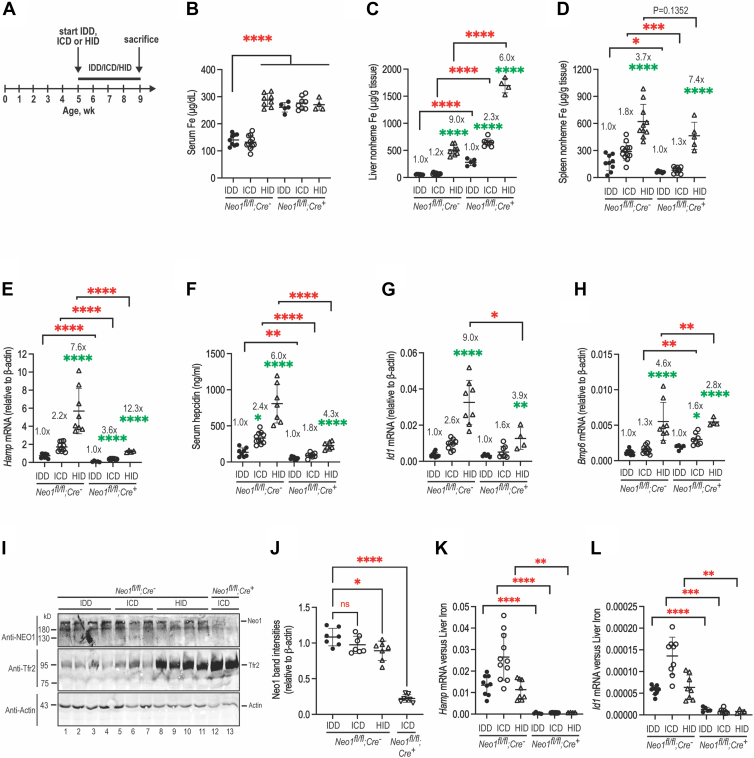
**Hepatic Neo1 is modestly reduced by increased bodily iron load, and ablation of hepatic Neo1 does not affect iron induction of hepatic hepcidin expression**. *A*, experimental design to examine the effects of bodily iron load on hepatic Neo1 expression and the effects of hepatic Neo1 on iron induction of hepcidin expression. Five-week-old WT (*Neo1*^*fl/fl*^;*Alb-Cre*^*-*^) and hepatic *Neo1*^−/−^ (*Neo1*^*fl/fl*^;*Alb-Cre*^*+*^) mice of both genders were fed an IDD, ICD, or HID diet for 4 weeks before euthanasia for analysis. All presented data in this figure are generated from male mice. *B*, serum iron assays. One-way ANOVA was used for analysis. *C*, liver nonheme iron assays. For this and other figures, the numbers represent the fold change per strain. *Green asterisks* represent one-way ANOVA analysis of the data per strain of mice relative to the corresponding IDD group. *Red asterisks* represent Two-tailed student-T test for mice fed the same iron diet. *D*, spleen nonheme iron assays. *E*, qRT-PCR analysis of *Hamp* mRNA levels in the liver. All qRT-PCR results are expressed as the amount relative to that of *β-actin* for each sample. *F*, serum hepcidin assay. *G* or *H*, qRT-PCR analysis of *Id1* and *Bmp6* mRNA levels in the liver. *I*, representative images of Western blot analysis for Neo1, Tfr2, and β-actin in the liver membrane preparations. *J*, quantification of Neo1 bands in I. The relative amounts to β-actin are presented. *K* or *L*, normalized hepatic *Hamp* and *Id1* mRNA levels to those of liver nonheme iron (*E*/*C* and *G*/*C*). Each group consists of at least four animals. The means ± SD are presented. ns, no statistical difference. ∗, *p* < 0.05; ∗∗, *p* < 0.01; ∗∗∗, *p* < 0.001; ∗∗∗∗, *p* < 0.0001. NEO1, neogenin; IDD, iron deficient diet.

Our earlier studies suggest that hepatic Neo1 acts as a scaffold to facilitate hepcidin expression *via* the Bmp-signaling pathway (22, 41). To determine whether hepatic Neo1 is involved in iron induction of hepcidin, we first tested whether its expression is regulated by changes of bodily iron load in WT (*Neo1*^*fl/fl*^;*Alb-Cre*^*-*^; *Tmprss6*^+/+^) mice. Five-week-old animals of both genders were fed an iron deficient diet (IDD; 2–6 ppm iron), an iron control diet (ICD; 48 ppm iron), or a high iron diet (HID; 5000 ppm iron) for 4 weeks before euthanasia for analysis (Fig. 8*A*). As expected, mice fed a HID diet displayed marked increases in serum iron, liver and spleen nonheme iron, hepatic *Hamp* mRNA, serum hepcidin, hepatic *Id1* mRNA, and *Bmp6* mRNA levels when compared with the ICD controls (Fig. 8, *B*–*H*; Fig. S5, *A*–*G*). The IDD groups exhibited significant decrease or trend decrease in liver and spleen nonheme iron, *Hamp* mRNA, serum hepcidin, hepatic *Id1* mRNA, and *Bmp6* mRNA levels (Fig. S5*H*; Fig. 8, *C*–*H*). The positive correlation between *Hamp* and *Id1* mRNA levels indicate that hepatic hepcidin expression is induced through the Bmp-signaling pathway (1, 67). Interestingly, we detected a modest decrease in Neo1 protein levels by increased bodily iron load (Fig. 8, *I* and *J*). No significant change of hepatic *Neo1* mRNA was observed between IDD, ICD, and HID groups (Fig. S5*I*). These observations suggest that increased bodily iron load facilitates the degradation of hepatic Neo1.

Next, we evaluated the iron regulation of hepcidin expression in *Neo1*^−/−^ (*Neo1*^*fl/fl*^;*Alb-Cre*^*+*^;*Tmprss6*^+/+^) mice. Similarly to WT counterparts, the liver nonheme iron levels in *Neo1*^−/−^ mice were decreased by feeding an IDD diet and increased by feeding a HID diet, except that the levels are proportionally higher (Fig. 8*C*; Fig. S5*B*). Different from WT mice, serum iron concentrations remained at constantly high levels regardless of bodily iron loads (Fig. 8*B*; Fig. S5*A*), similar to *Hjv*^−/−^ mice (65). Interestingly, *Hamp* mRNA, serum hepcidin, *Id1* mRNA, and *Bmp6* levels were all parallelly elevated by increased bodily iron load in the HID groups and lowered by reduced bodily iron load in the IDD groups (Fig. 8, *E*–*H*; Fig. S5, *D*–*G*). These results indicate that *Neo1*^−/−^ mice retain an intact iron sensing mechanism to induce hepcidin *via* the Bmp-signaling pathway. The parallel increases in hepatic *Bmp6* mRNA levels to bodily iron load (Fig. 8, *C* and *H*; Fig. S5, *B* and *G*) suggest that the iron induction of hepcidin in *Neo1*^−/−^ mice is likely attributed to the increased Bmp6 expression. However, even though the *Hamp* and *Id1* mRNA was positively increased by iron in *Neo1*^−/−^ mice, they were less responsive than WT counterparts when normalized to liver iron concentration (Fig. 8, *K* and *L*; Fig. S5, *J* and *K*). Normalization to liver iron concentration is often used to evaluate the impairment of hepcidin induction by iron (68, 69). These findings indicate that hepatic Neo1 is an essential component of the Bmp-signaling pathway. Together, these observations suggest that the major function of hepatic Neo1 is to set the basal levels of hepcidin expression.

### Lack of both Neo1 and Tmprss6 in the liver does not affect iron induction of hepcidin expression

**Figure 9:**
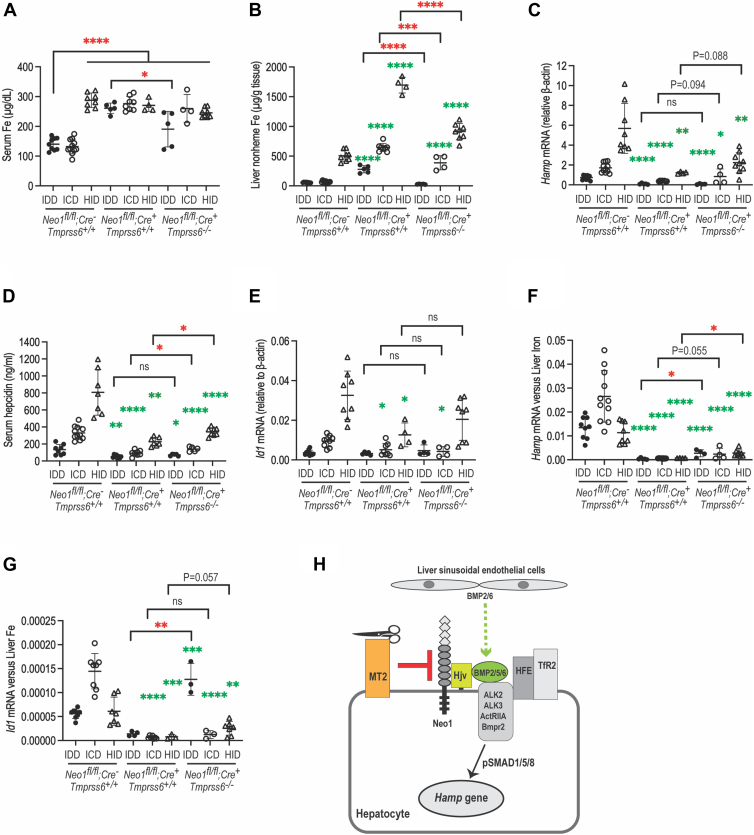
**Ablation of both Tmprss6 and hepatic Neo1 does not affect iron induction of hepcidin expression**. Five-week-old *Neo1*^*fl/fl*^;*Alb-Cre*^*+*^;*Tmprss6*^−/−^ mice of both genders were fed an IDD, ICD, or HID for 4 weeks before euthanasia for analysis. These animal models were generated in the same setting of studies as described in the legend to Figure 8 for *Neo1*^*fl/fl*^;*Alb-Cre*^*-*^ and *Neo1*^*fl/fl*^;*Alb-Cre*^*+*^ mice. All presented data in this figure are generated from male mice. *A*, serum iron assay. One-way ANOVA was used for analysis. *B*, liver nonheme iron assay. *Green asterisks* represent one-way ANOVA analysis of the data from mice fed the same iron diet relative to the corresponding WT (*Neo1*^*fl/fl*^;*Alb-Cre*^*-*^;*Tmprss6*^+/+^) controls. *Red asterisks* represent Two-tailed student-T test between *Neo1*^*fl/fl*^;*Alb-Cre*^*+*^;*Tmprss6*^+/+^ and *Neo1*^*fl/fl*^;*Alb-Cre*^*+*^;*Tmprss6*^−/−^ mice fed the same iron diet. *C*, qRT-PCR analysis of *Hamp* mRNA levels in the liver. All qRT-PCR results are expressed as the amount relative to that of *β-actin* for each sample. *D*, serum hepcidin assay. *E*, qRT-PCR analysis of *Id1* mRNA levels in the liver. *F* or *G*, normalized hepatic *Hamp* and *Id1* mRNA levels to those of liver nonheme iron (C/B and E/B). Each group consists of at least four animals. The means ± SD are presented. ns, no statistical difference. ∗, *p* < 0.05; ∗∗, *p* < 0.01; ∗∗∗, *p* < 0.001; ∗∗∗∗, *p* < 0.0001. *H*, a model for Mt2 suppression of hepcidin expression in hepatocytes. IDD, iron deficient diet; NEO1, neogenin.

Since previous studies suggest that the function of Mt2 is also to set the basal levels of hepcidin (52), we next tested the effects by ablation of both *Neo1* and *Tmprss6*. The *Neo1*^−/−^;*Tmprss6*^−/−^ mice with different iron loads were generated in the same setting as described above for *Neo1*^−/−^ and WT littermates. When compared with *Neo1*^−/−^ mice, feeding an IDD diet for 4 weeks resulted in a modest reduction of serum iron in *Neo1*^−/−^;*Tmprss6*^−/−^ mice (Fig. 9A; Fig. S6*A*). The liver nonheme iron levels in all groups of *Neo1*^−/−^;*Tmprss6*^−/−^ mice were proportionally lower than those in *Neo1*^−/−^ mice, but the levels in ICD and HID groups remained substantially higher than those in the corresponding WT animals (Fig. 9*B*; Fig. S6*B*). The splenic iron levels in *Neo1*^−/−^;*Tmprss6*^−/−^ mice were also lower than those in *Neo1*^−/−^ and WT littermates (Fig. S6*C*) implying no bodily iron redistribution. These dynamic iron studies suggest that a simultaneous ablation of *Tmprss6* and *Neo1* can attenuate, but not prevent, the increased iron load by ablation of hepatic *Neo1* alone.

Similar to WT and *Neo1*^−/−^ mice, hepatic *Hamp* mRNA and serum hepcidin levels in *Neo1*^−/−^;*Tmprss6*^−/−^ mice were reduced by iron depletion in the IDD groups and elevated by increased bodily iron load in the HID groups (Fig. 9, *C* and *D*; Fig. S6, *D* and *E*). A comparable pattern was also observed for *Id1* mRNA levels (Fig. 9*E*; Fig. S6*F*). These results indicate that lack of both Neo1 and Mt2 fails to affect iron induction of hepcidin *via* the Bmp-signaling pathway. Although *Hamp* mRNA and serum hepcidin levels in all groups of *Neo1*^−/−^;*Tmprss6*^−/−^ mice were significantly lower than those in the corresponding WT counterparts, *Neo1*^−/−^;*Tmprss6*^−/−^ mice displayed modestly higher levels of *Hamp* mRNA and serum hepcidin in the ICD and HID groups than those in *Neo1*^−/−^ mice (Fig. 9, *C* and *D*; Fig. S6, *D* and *E*). These data suggest that the relatively lower bodily iron load in *Neo1*^−/−^;*Tmprss6*^−/−^ mice than *Neo1*^−/−^ mice results from the elevated hepcidin expression. No significant increases in hepatic *Bmp6* mRNA levels were detected in *Neo1*^−/−^;*Tmprss6*^−/−^ mice (Fig. S6*G*). These results suggest that the elevated hepcidin expression in *Neo1*^−/−^;*Tmprss6*^−/−^ mice is likely attributed to the lack of Mt2. Further analyses by normalization to liver iron concentration (Fig 9, *F* and *G*; Fig. S6, *H* and *I*) imply that in response to iron challenge, the relatively higher hepcidin levels in *Neo1*^−/−^;*Tmprss6*^−/−^ mice than *Neo1*^−/−^ mice likely result from the elevated Bmp-signaling. Together, the above data support the idea that lack of both hepatic Neo1 and Mt2 does not affect iron induction of hepcidin and that hepatic Neo1 is not the sole target of Mt2 to suppress hepcidin expression.

## Discussion

In this study, we investigated the roles of Neo1-Mt2 association in hepcidin expression. We found that Mt2 acts to stabilize hepatic Neo1 in the liver. Our *in vivo* data indicate that Mt2 suppression of hepcidin is achieved largely by inhibiting the Neo1-facilitated Bmp-signaling and suggest that hepatic Neo1 is a major target of Mt2. Additional studies revealed that hepatic Neol is reduced modestly by increased bodily iron loads and imply that the major function of Neo1 is to set the basal level of hepcidin expression.

The *in vivo* results for the roles of Mt2’s proteolytic activity in mice are in contrast to the *in vitro* studies in hepatoma cells. Most of the earlier studies for the function of Mt2 were conducted by transient co-transfection in cell lines and show that Mt2 cleaves multiple key hepcidin-inducing components (51, 53, 54, 70, 71). Overexpression of proteases can cause promiscuous non-physiological cleavage. By employing a similar approach in this study, we found that the transiently transfected Mt2 is also able to markedly reduce the co-transfected Neo1 levels in hepatoma cells *via* its proteolytic activity.

Our *in vivo* studies demonstrated the limitations of utilizing the obtained data from cell lines to predict the biological functions of Mt2. Distinct from the findings *in vitro*, we found that lack of Mt2 reduces, rather than increases, Neo1 levels in the liver. Importantly, this Neo1 reduction is fully corrected by expressing exogenously administered fMt2 to suppress hepcidin expression. Interestingly, hepatic Neo1 levels are positively correlated with those of expressed fMt2 in the liver. These observations indicate that Mt2 does not inactivate Neo1 by cleavage *in vivo* and suggest that it stabilizes Neo1 in the liver. This idea is supported by the previous studies showing that the suppression of hepcidin expression by Mt2 is not associated with the decrease in hepatic Hjv (55, 72, 73). MT2 interacts with NEO1 and HJV (29, 53). Additionally, in contrast to the observations that Mt2 cleaves and reduces Tfr2 in transfected cells (51), we found a positive correlation of hepatic Tfr2 levels with those of Mt2 and serum iron. This result also suggests that Mt2 does not cleave its binding partners *in vivo*. We speculate that the discrepancies between *in vitro* and *in vivo* studies arise because cell lines do not express many of the genes that are involved in iron homeostasis in hepatocytes (22). Together, all these data support the idea that Mt2 suppresses hepcidin expression by binding and sequestering its partners, rather than through its proteolytic activity.

We further investigated the relationship between Mt2 and Neo1 in hepcidin expression in mice. Results show that with a steady iron supply, mice deficient for both Mt2 and hepatic Neo1 displayed comparable extents of hepcidin mRNA reduction and iron overload as seen in *Neo1*^−/−^ mice and complete abolishment of the inappropriately high hepcidin expression and iron deficiency anemia as observed in *Tmprss6*^−/−^ mice. These observations suggest that Mt2 regulates iron homeostasis by suppressing the Neo1-facilitated hepcidin expression. Since Mt2 acts to stabilize hepatic Neo1 in the liver, we speculate that Mt2 inhibits the function of hepatic Neo1 likely by Mt2-Neo1 association as suggested in our earlier study (55). Alternatively, it is also plausible that the decreased *Hamp* mRNA and iron overload in *Neo1*^−/−^;*Tmprss6*^−/−^ mice results from the indirectly disrupted Hjv function due to the lack of Neo1. Hjv is a robust inducer of hepcidin expression (8, 28), and Hjv induction of hepcidin requires its association with Neo1 (22, 30). Mice deficient for both *Tmprss6* and *Hjv* display markedly decreased *Hamp* mRNA levels and systemic iron overload similar to mice deficient for *Hjv* alone (50, 56). Thus, Mt2 suppression of hepcidin could be accomplished by blocking the Neo1/Hjv-mediated signaling.

We also determined whether hepatic Neo1-facilitated signaling is the only target of Mt2 by comparing *Neo1*^−/−^;*Tmprss6*^−/−^ and *Neo1*^−/−^ mice, which were fed different iron diets. Interestingly, we found that in the absence of hepatic Neo1, a simultaneous lack of Mt2 is still able to modestly increase hepcidin expression and to reduce liver nonheme iron content accordingly. However, the extent of changes is much less than those seen in *Tmprss6*^−/−^ mice that lack Mt2 alone. No iron deficiency anemia was observed in mice lacking both *Neo1* and *Tmprss6*. These observations indicate that in the absence of hepatic Neo1, Mt2 is still able to suppress hepcidin expression but to a limited extent and suggest that hepatic Neo1 is not the only target of Mt2. An earlier study reports that the action of Mt2 also depends on the presence of Bmp6 (74). Hjv is a BMP coreceptor and it utilizes BMP6 to induce hepcidin (16). Based on these and other studies (16, 50, 56, 74), we speculate that Mt2 suppresses hepcidin likely by inhibiting the function of a complex that contains at least Neo1, Hjv, and Bmp6, rather than a single component (Fig. 9*H*).

The liver possesses an elegant machinery to positively regulate hepcidin expression in response to increased iron load *via* the BMP-signaling pathway (2, 13, 21, 57, 58, 59, 60, 61). Earlier studies show that neither Hjv nor Mt2 is required for iron induction of hepcidin and that Hjv and Mt2 act to set the basal levels of hepcidin (55, 65, 75). Here our data suggest that the major function of hepatic Neo1 is also to set the basal hepcidin expression to appropriately high levels, similarly to Hjv. Although we detected a modest decrease in Neo1 protein levels in response to increased bodily iron load in WT mice, the underlying mechanism and its physiological significance in hepcidin expression remain to be determined. Additionally, we found that lack of both Neo1 and Mt2 failed to blunt iron induction of hepcidin in mice. Since HJV, NEO1 and MT2 can form a tertiary complex *in vitro* (29), we speculate that they likely act together as a complex to set hepcidin expression to physiological levels and that iron regulation of hepcidin is achieved by the changes of hepatic Tfr2 levels and Bmp2/6 supply as previously indicated (7, 16, 17, 20, 62, 63, 64).

The clinical significance of our findings is potentially important. Recent studies have identified MT2 as a target for the treatment of iron overload disorders (76, 77, 78). Our data suggest that the attempt to inhibit the proteolytic activity of MT2 by using specific inhibitors (79, 80, 81) might not be able to block the function of MT2 *in vivo*. This idea is also supported by the clinical studies showing that at least three disease-causing mutants (R271Q, T287 N, and G442 R) in the CUB domains of MT2 (Fig. 1*D*) behave similarly to WT MT2 with respect to cell surface localization and substrate cleavage (53, 70, 71, 82). Rather, our findings support that either depletion of MT2 or disruption of substrate interaction would be the appropriate strategy to achieve this goal. Given that numerous homozygous mutations in different subdomains of MT2 (including SEA, CUB1, CUB2, L, and S/P; Fig. 1*D*) all disrupt the function of MT2 and cause iron-refractory iron-deficiency anemia (70, 71, 83, 84, 85, 86, 87), these earlier studies imply that the entire ectodomain of MT2 is involved in the association with its binding partners. However, it remains to be determined how these interactions occur.

In summary, our data demonstrated that Mt2 suppression of hepcidin expression requires hepatocyte Neo1. In conjunction with earlier studies (16, 50, 56, 74), our data support the model in which Mt2 acts through association with Neo1/Hjv/Bmp6 to control Bmp-signaling and hepcidin expression (Fig. 9*H*).

## Experimental procedures

### cDNA constructs

We purchased murine Neo1 ORF (NM_008684) with a C-terminal FLAG/MYC epitope (fNeo1) in pCMV6 vector (#MR226235) and murine Mt2 ORF (NM_027902.1) with a C-terminal FLAG/MYC epitope (fMt2) in pCMV6 vector (#MR210781) from OriGene Technologies Inc. The entire sequences were validated by DNA sequencing. The pCMV6-fMt2^*mask*^ construct that lacks the coding sequence for the proteolytic domain was generated in our previous studies (51). fMt2 and fMt2^*mask*^ constructs were subcloned into an AAV8 construct containing a strong liver-specific promoter as described in our previous study (65). The liver-specific promoter is a combination of two copies of a human α1-microglobulin/bikunin enhancer and the promoter from the human thyroid hormone-binding globulin gene. AAV8-fMt2 and fMt2^*mask*^ viral vectors were generated at the Molecular Virology Support Core, OHSU.

### Cell line and transfection

We obtained Hep3B cells from ATCC (#HB-8064). We used Hep3B cells for transient co-transfection of pCMV6-fNeo1 with pEGFP-N1 (EGFP) or pCMV6-fMt2 construct DNA at 1:1 ratio with Lipofectamine-3000 (Invitrogen) to determine the cleavage of Neo1 by Mt2. At ∼36 h post-transfection, aprotinin (AP; G Biosciences) was added to the culture medium at 5 μM to inhibit the proteolysis of Mt2. After additional ∼16 h of incubation, cell surface proteins were biotinylated on ice as previously described (41). The biotinylated proteins and ∼10% fraction of whole cell extracts were subjected to SDS-PAGE and immunodetection by using an HRP-coupled mouse anti-FLAG M2 IgG (Sigma) or by using rabbit anti-human NEO1 (29), mouse anti-Na^+^K^+^ ATPase (Santa Cruz Biotechnology; sc-21712), mouse anti-β-actin (Sigma), and the corresponding secondary antibodies. All images were captured by using the c600 Western blot imaging system (Azure Biosystems, Inc). The intensities of specific bands were quantified by using the ImageJ software (imagej.net/ij/download.html). The amounts relative to Na^+^K^+^ ATPase are presented.

### Animal studies

All animal procedures were approved by OHSU/DCM. We generated heterozygous *Tmprss6*^*+/−*^ mutant mice and homozygous hepatocyte-specific conditional *Neo1* knockout (*Neo1*^*fl/fl*^;*Alb-Cre*^*+*^) mice on a C57BL/6J background in our previous studies (22, 52). We generated *Neo1/Tmprss6*-double knockout (*Neo1*^*fl/fl*^;*Alb-Cre*^*+*^;*Tmprss6*^−/−^), *Tmprss6*-single knockout (*Neo1*^*fl/fl*^;*Alb-Cre*^*-*^;*Tmprss6*^−/−^), *Neo1*-single knockout (*Neo1*^*fl/fl*^;*Alb-Cre*^*+*^;*Tmprss6*^+/+^), and WT (*Neo1*^*fl/fl*^;*Alb-Cre*^*-*^;*Tmprss6*^+/+^) littermates by crossing *Neo1*^*fl/fl*^;*Alb-Cre*^*−/−*^;*Tmprss6*^+/−^ male and *Neo1*^*fl/fl*^;*Alb-Cre*^*+/−*^;*Tmprss6*^+/−^ female on a C57BL/6J background. All mice were fed a PicoLab Laboratory Rodent Diet-5L0D containing 240-ppm iron (LabDiet). Mice of both genders were euthanized for analysis at the indicated age in the figure legends.

To study the roles of Neo1 and Neo1/Mt2 in iron regulation of hepcidin expression, five-week-old *Neo1*^*fl/fl*^;*Alb-Cre*^*+*^, *Neo1*^*fl/fl*^;*Alb-Cre*^*+*^;*Tmprss6*^−/−^, and their corresponding WT littermates of both genders were randomly divided into three groups and fed an iron deficient diet (IDD; 2–6 ppm iron; TD.110669), an ICD; 48 ppm iron; TD.09488), or a HID; 5000 ppm iron; TD.140464) for 4 weeks prior to euthanasia for analysis.

To determine the effects of Mt2 on hepatocyte Neo1, we intraperitoneally administered AAV8-fMt2 and AAV8-fMt2^*mask*^ viral vectors into eight-week-old *Tmprss6*^*−/−*^ littermates of both genders on a mixed B6/129S background and eight-week-old WT mice of both genders on a 129S background as described in our earlier studies (51, 54). The administered dosages for AAV8-fMt2 or AAV8-fMt2^*mask*^ vectors are described in figure legends. Injection of sterilized PBS vehicle was included as controls. Three weeks later, mice were euthanized for analysis. Blood was collected by cardiac puncture for serum iron, serum hepcidin, and hematology analysis. Liver and spleen were rapidly removed, snap-frozen in liquid nitrogen and then stored at −80 °C for qRT-PCR, Western blot, and tissue nonheme iron assays. Age, gender, and background-matched WT littermates for *Tmprss6*^*−/−*^ mice were included as additional controls. The AAV8 viral vector alone had no effect on iron homeostasis in mice (30, 88).

### Blood parameters, serum iron, and serum hepcidin assays

Blood parameters were analyzed by using the Hemavet 950 (Drew Scientific). Serum iron and hepcidin concentrations were detected by using the Pointe Iron/TIBC Reagent Set (Pointe Scientific) and the Hepcidin-Murine Compete ELISA kit (Intrinsic Lifesciences), respectively.

### Tissue nonheme iron assays

Tissue nonheme iron levels were determined as previously described (89) with the following modifications: 50 to 150 mg wet tissues were digested in 250 to 750 μl of acid buffer at 65 °C for 72 h. The supernatant was collected by centrifugation at 10,000g for 5 min, followed by addition of chromogen (1.86 mM bathophenanthroline sulfonate, 143 mM thioglycolic acid in water) and OD measurement at 535 nm. Each sample was measured twice in triplicate. Iron concentration is expressed as micrograms of iron per gram of wet tissue.

### qRT-PCR

Total RNA from mouse liver tissues was extracted using a NucleoSpin RNA kit (Macherey-Nagel). cDNA was synthesized using Oligo deoxythymidine primers (Invitrogen) and M-MLV reverse transcriptase (Invitrogen). qRT-PCR analysis of *Neo1*, *Hamp*, *Id1*, *Bmp6*, *Tmprss6*, and *β-actin* transcripts was carried out in triplicate on each sample using the Power SYBR Green PCR master mix (Applied Biosystems) and run on the QuantStudio 12K Flex qPCR System (Thermo Fisher Scientific). All primer sets used in these studies (Tables S1) were validated against the reference primers *(β-actin*) to ensure equal efficiencies of amplifications. The results are expressed as the amount relative to that of β-actin for each sample.

### Immunodetection

Liver membrane fractions were prepared as previously described (72). Protein extracts (∼250 μg) from the liver membrane preparations were separated by using SDS-PAGE under reducing conditions. Endogenous Neo1, Tfr2, Hjv, and β-actin were detected by using rabbit anti-human NEO1 fibronectin III 1 to 6 (29), rabbit anti-Tfr2 (90), mouse anti-Hjv (55), mouse anti-β-actin (Sigma), and the corresponding secondary antibodies or by HRP-coupled mouse anti-FLAG M2 IgG (Sigma). All images were captured by using the c600 Western blot imaging system (Azure Biosystems, Inc). The intensities of specific bands were quantified by using the ImageJ software. The amounts relative to β-actin are presented.

### Statistical analysis

Two-tailed student-T test was used to compare two sets of data. One-way ANOVA and Tukey’s post-test were used for multiple comparisons.

## Data availability

Raw data are available from the corresponding author on reasonable request.

## Supporting information

This article contains supporting information.

## Conflict of interest

The authors declare that they have no conflicts of interest with the contents of this article.
