# Supplementary material for: Matriptase-2-mediated suppression of hepatic hepcidin expression in mice requires hepatocyte neogenin
Source: J Biol Chem. 2026 Jan 12;302(3):111142. doi: 10.1016/j.jbc.2026.111142 (PMC12887391; doi:10.1016/j.jbc.2026.111142)
Supplement: Supplementary Material [file mmc1.pdf]

Matriptase-2-mediated suppression of hepatic hepcidin expression in mice requires hepatocyte neogenin

Caroline A. Enns, Shall Jue, and An-Sheng Zhang

List of supporting information:

Table S1
Figure S1
Figure S2
Figure S3
Figure S4
Figure S5
Figure S6

***Supplemental* Table S1. List of mouse-specific primers used for qRT-PCR analysis**

Gene	Forward primer	Reverse primer
Alk2	5'-TGTGAAGGCCAGCAGTGTTTT-3'	5'-CCCCTGCTCATAAACCTGAAAG-3'
Alk3	5'-TGCCCAGTGACCCATCCTA-3'	5'-TCGAAGACATTCATCGCTGTTC-3'
β-actin	5'-CTGCCTGACGGCCAGGT-3'	5'-TGGATGCCACAGGATTCCAT-3'
Bmp6	5'-AGCACAGAGACTCTGACCTATTTTTG-3'	5'-CCACAGATTGCTAGTTGCTGTGA-3'
Hamp	5'-CACCAACTTCCCCATCTGCATCTT-3'	5'-GAGGGGCTGCAGGGGTGTAGAG-3'
Hfe	5'-TCTGGGACAGCAAGTGCCTAC-3'	5'-GGCATCCAGTGGTTGGTTGT-3'
Id1	5'-ACCCTGAACGGCGAGATCA-3'	5'-TCGTCGGCTGGAACACATG-3'
Neo1	5'-CGAGCTGCGTGCAAATCA-3'	5'-ATCCATAGGTCTGGAGGCTTCAC-3'
Tfr2	5'-GCTGGGACGGCGGTGACTT-3'	5'-GAGTTGTCCAGGCTCACGTACA-3'
Tmprss6	5'-TTGCTGGTCTTGGCTGCGCT-3'	5'-AATGACGGTTGAGCACCCGGAG-3'

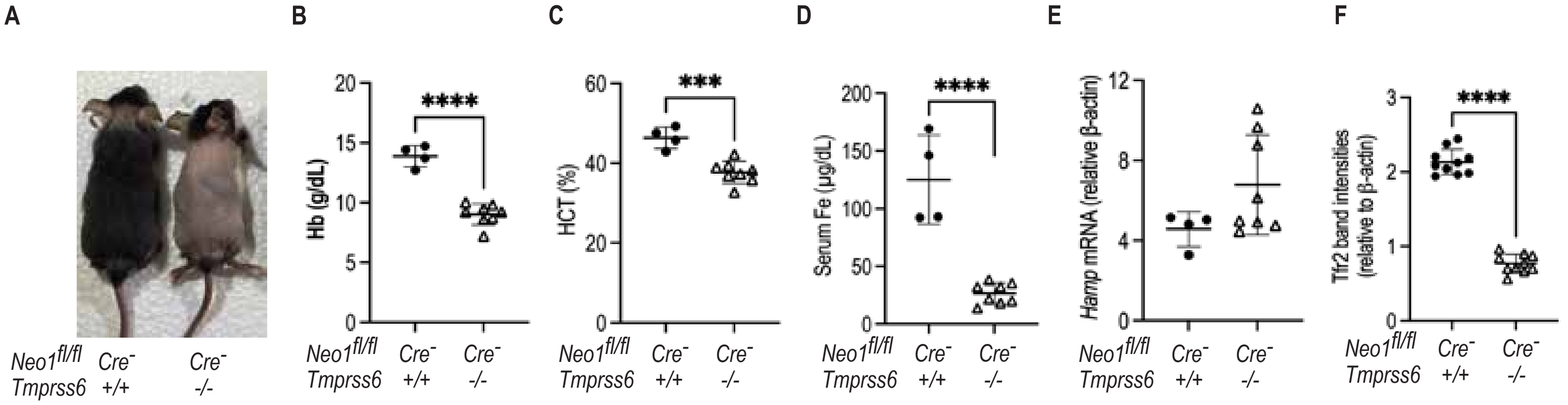

Supplemental **Figure S1.** *Global ablation of Tmprss6 causes iron deficiency anemia in mice*. **A)** A representative image of wild-type (*Neo1*$^{fl/fl}$;*Alb-Cre*$^{-}$;*Tmprss6*$^{+/+}$) and *Tmprss6*$^{-/-}$ (*Neo1*$^{fl/fl}$;*Alb-Cre*$^{-}$;*Tmprss6*$^{-/-}$) mice at the time of euthanasia. **B/C)** Blood parameter analysis: hemoglobin (Hb) and hematocrit (HCT). **D)** Serum iron assay. **E)** qRT-PCR analysis of hepatic *Hamp* mRNA. **F)** Quantification of Tfr2 bands in **Figure 3D**. The relative amounts to β-actin are presented. Two-tailed student T-test was used for analysis. ***, $P<0.001$; ****, $P<0.0001$.

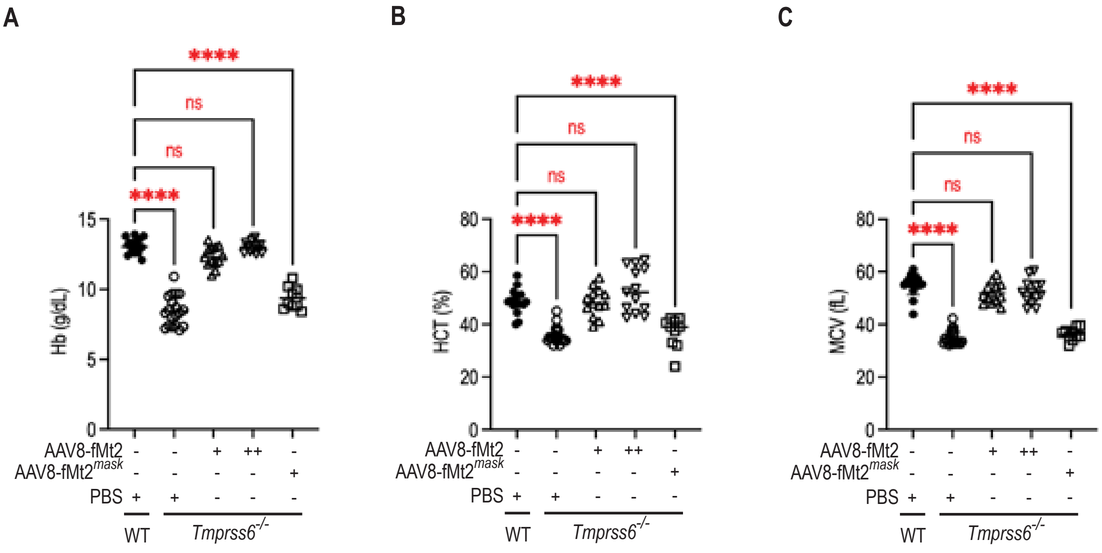

Supplemental **Figure S2.** Blood parameter analysis for mice as described in the legend to **Figure 4**: **A)** hemoglobin (Hb). **B)** Hematocrit (HCT). **C)** Mean corpuscular volume (MCV). ns, no statistical difference. ****, P<0.0001.

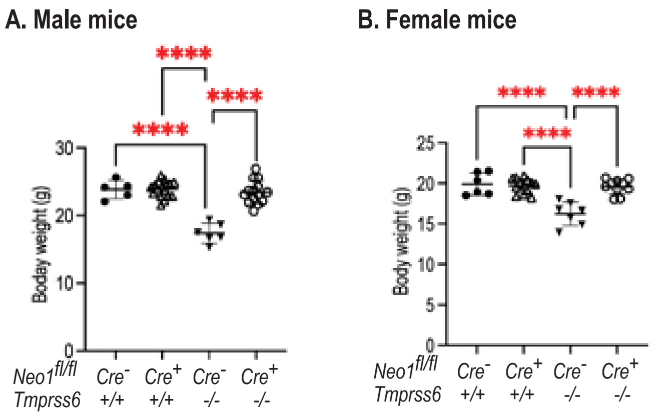

Supplemental **Figure S3.** Body weight of 8-week-old *Neo1*$^{fl/fl}$;*Alb-Cre*$^{-}$;*Tmprss6*$^{+/+}$, *Neo1*$^{fl/fl}$;*Alb-Cre*$^{+}$;*Tmprss6*$^{+/+}$, *Neo1*$^{fl/fl}$;*Alb-Cre*$^{-}$;*Tmprss6*$^{-/-}$, and *Neo1*$^{fl/fl}$;*Alb-Cre*$^{+}$;*Tmprss6*$^{-/-}$ mice of male and female in **Figure 6**. Each group consists of at least five mice. All data shown are means ± SD. One-way ANOVA was used for analysis. ****, P<0.0001.

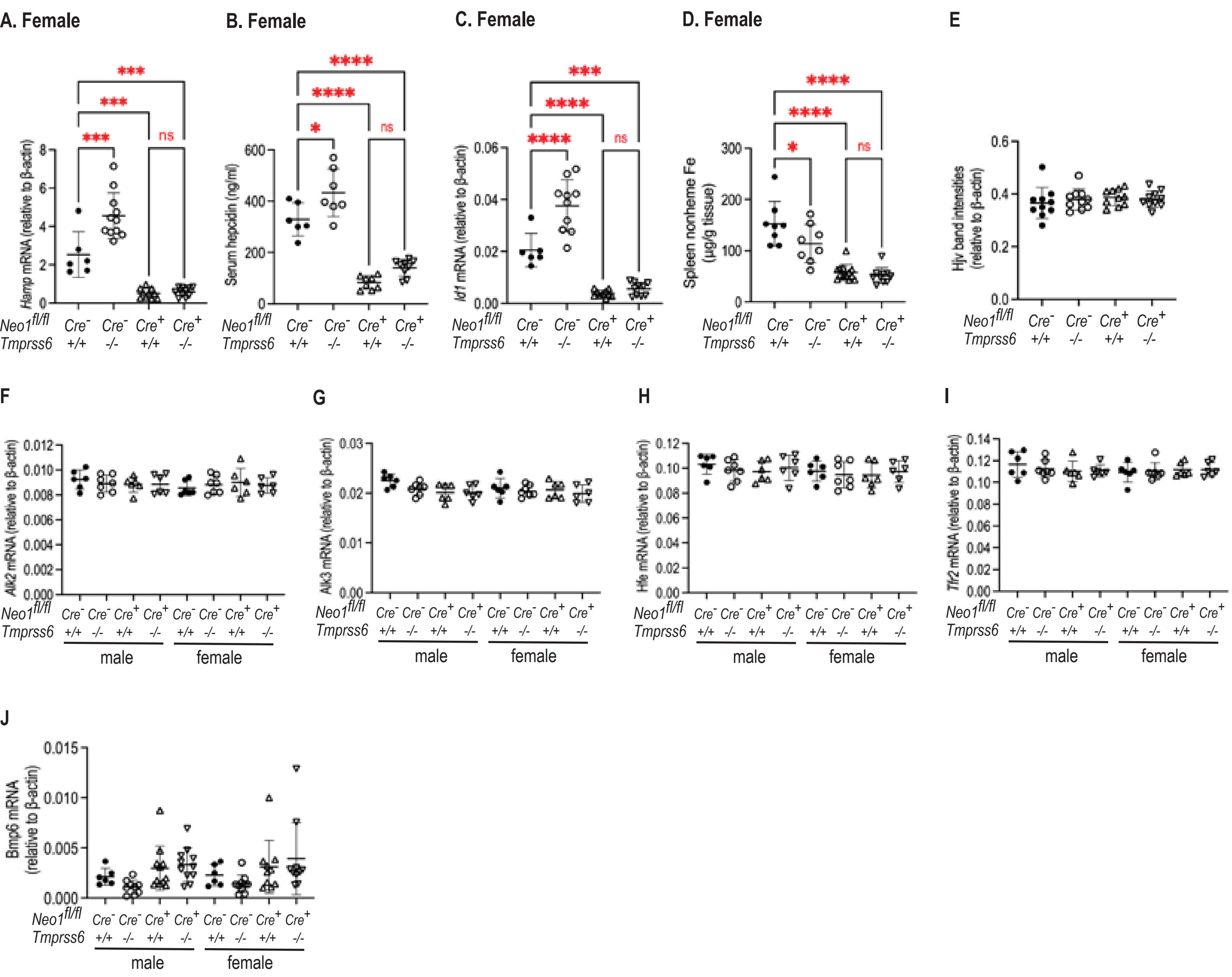

***Supplemental* Figure S4.** *Ablation of both Tmprss6 and hepatic Neo1 reduces hepatic hepcidin expression.* **A)** qRT-PCR analysis of *Hamp* mRNA in the liver of 8-week-old female *Neo1*$^{fl/fl}$;*Alb*-Cre^{-};*Tmprss6*$^{+/+}$, *Neo1*$^{fl/fl}$;*Alb*-Cre^{-};*Tmprss6*$^{-/-}$, *Neo1*$^{fl/fl}$;*Alb*-Cre^{+};*Tmprss6*$^{+/+}$, and *Neo1*$^{fl/fl}$;*Alb*-Cre^{+};*Tmprss6*$^{-/-}$ mice as described in the legend to **Figure 6**. **B)** Serum hepcidin assay of female mice. **C)** qRT-PCR analysis of *Id1* mRNA in the liver of female mice. **D)** Spleen nonheme iron assay of female mice. **E)** Quantification of Hjv bands in **Figure 7E**. **F-J)** qRT-PCR analysis of *Alk2*, *Alk3*, *Hfe*, *Tfr2*, and *Bmp6* mRNA in the liver of male and female mice. All results are expressed as the amount relative to that of β-actin for each sample. All data shown are means ± SD. One-way ANOVA was used for analysis. ns, no statistical difference. *, P<0.05; ***, <0.001; ****, P<0.0001.

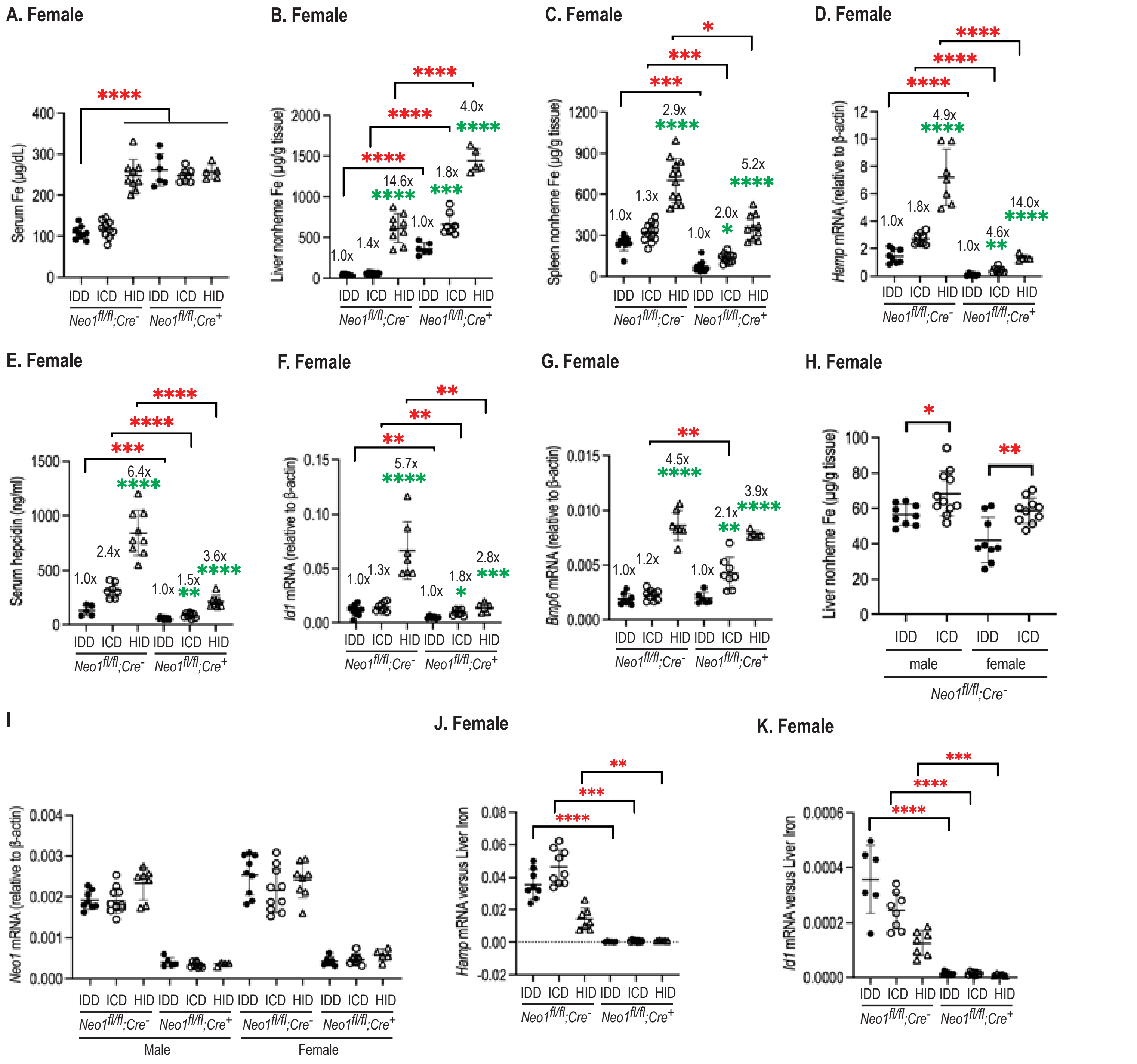

Supplemental **Figure S5.** *Hepatic Neo1 mRNA levels are not altered by the levels of bodily iron load, and ablation of hepatic Neo1 does not affect iron induction of hepatic hepcidin expression.* All studies are described in the legend to **Figure 8**. **A)** Serum iron assays of female mice. **B)** Liver nonheme iron assays of female mice. For this and other figures, the numbers represent the fold change per strain. Green asterisks represent one-way ANOVA analysis of the data per strain of mice relative to the corresponding IDD group. Red asterisks represent Two-tailed student-T test for mice fed the same iron diet. **C)** Spleen nonheme iron assays of female mice. **D)** qRT-PCR analysis of *Hamp* mRNA levels in the liver of female. All qRT-PCR results are expressed as the amount relative to that of β-actin for each sample. **E)** Serum hepcidin assay of female mice. **F/G)** qRT-PCR analysis of *Id1* and *Bmp6* mRNA levels in the liver of female mice. **H)** Liver nonheme iron assays in wild-type (*Neo1*$^{fl/fl}$;*Alb-Cre*$^{-}$;*Tmprss6*$^{+/+}$) mice fed an iron deficient diet (IDD; 2-6 ppm iron) and an iron control died (ICD; 48 ppm iron) in **Figure 8C**. Two-tailed student T-test was used for analysis. **I)** qRT-PCR analysis of *Neo1* mRNA in the liver of mice in **Figure 8**. **J/K)** Normalized hepatic *Hamp* and *Id1* mRNA levels to those of liver nonheme iron (**D/B** and **F/B**). Each group consists of at least 4 animals. *, P<0.05; **, P<0.01; ***, P<0.001; ****, P<0.0001.

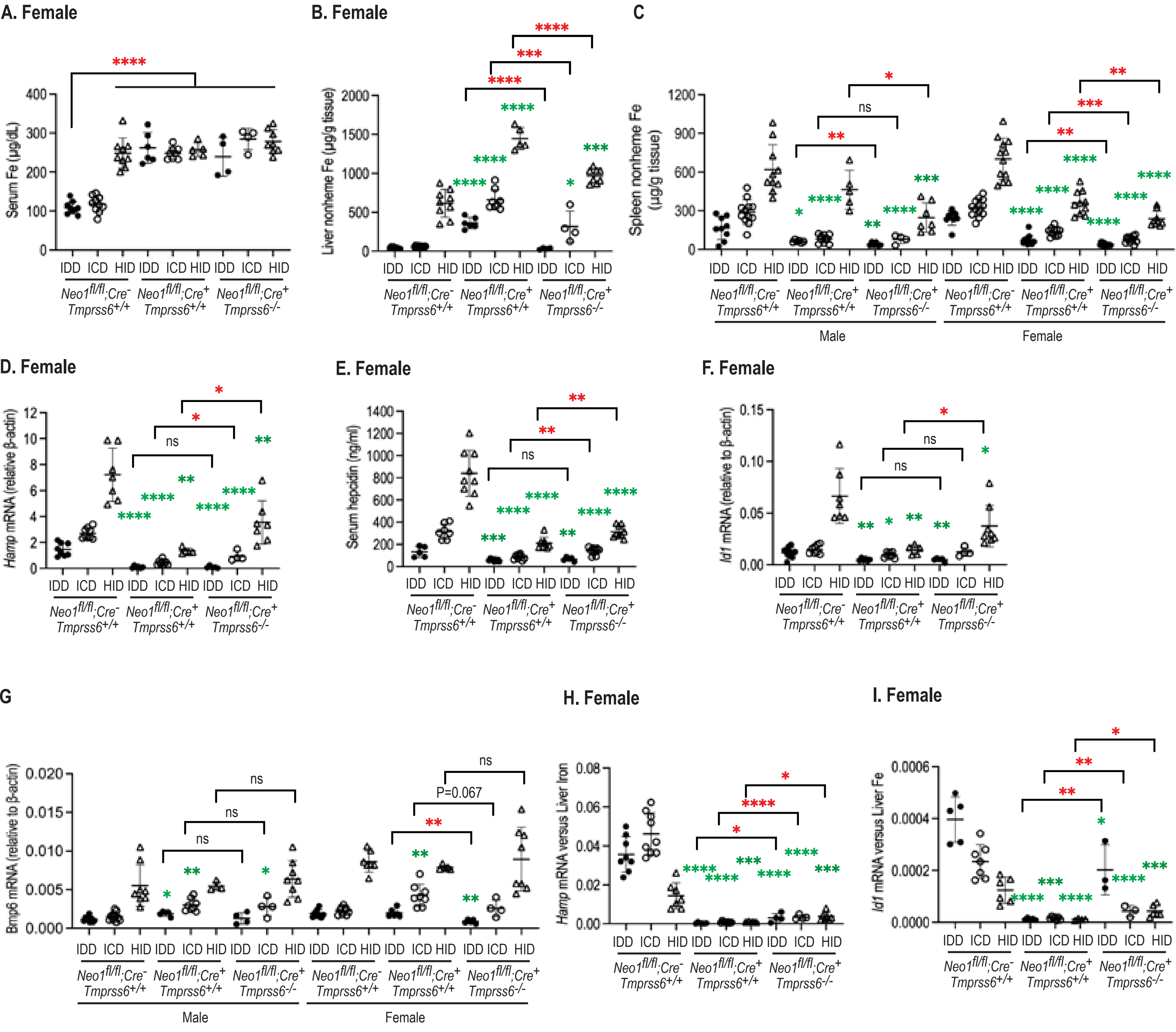

***Supplemental* Figure S6**. *Ablation of both Tmprss6 and hepatic Neo1 does not affect iron induction of hepcidin expression.* All studies are described in the legend to **Figure 9**. **A)** Serum iron assay of female mice. **B)** Liver non-heme iron assay of female mice. Green asterisks represent One-way ANOVA analysis of the data from mice fed the same iron diets relative to the corresponding wild-type (*Neo1*$^{fl/fl}$;*Alb*-Cre^{-};*Tmprss6*$^{+/+}$) controls. Red asterisks represent Two-tailed student-T test between *Neo1*$^{fl/fl}$;*Alb*-Cre^{+};*Tmprss6*$^{+/+}$ and *Neo1*$^{fl/fl}$;*Alb*-Cre^{+};*Tmprss6*$^{-/-}$ fed the same iron diet. **C)** Spleen non-heme iron assay of both genders. **D)** qRT-PCR analysis of *Hamp* mRNA levels in the liver of female mice. **E)** Serum hepcidin assay of female mice. **F)** qRT-PCR analysis of *Id1* mRNA levels in the liver of female mice. **G)** qRT-PCR analysis of *Bmp6* mRNA levels in the liver of male and female mice. **H/I)** Normalized hepatic *Hamp* and *Id1* mRNA levels to those of liver nonheme iron (**D/B** and **F/B**) for female animals. Each group consists of at least 4 animals. ns, no statistical difference. *, P<0.05; **, P<0.01; ***, P<0.001; ****, P<0.0001.
